# The Synergistic Effect of Glucagon-Like Peptide-1 and Chamomile Oil on Differentiation of Mesenchymal Stem Cells into Insulin-Producing Cells

**DOI:** 10.22074/cellj.2020.6325

**Published:** 2019-07-31

**Authors:** Saeid Saghahazrati, Seyed Abdulmajid Ayatollahi, Farzad Kobarfard, Bagher Minaii Zang

**Affiliations:** 1 Phytochemistry Research Center, Shahid Beheshti University of Medical Sciences, Tehran, Iran; 2 Department of Chemistry, Richardson College for The Environmental Science Complex, The University of Winnipeg, Winnipeg, Canada; 3 Department of Pharmacognosy, School of Pharmacy, Shahid Beheshti University of Medical Sciences, Tehran, Iran; 4 Department of Medicinal Chemistry, Shahid Beheshti School of Pharmacy, Tehran, Iran; 5 Department of Histology, Faculty of Medicine, Tehran University of Medical Sciences, Tehran, Iran

**Keywords:** Chamomile Oil, Differentiation, Glucagon-Like Peptide-1, Insulin-Producing Cells, Mesenchymal Stem Cell

## Abstract

**Objective:**

Glucagon-like peptide-1 (GLP-1) has attracted tremendous attention for treatment of diabetes. Likewise, it seems that active ingredients of chamomile oil might have anti-diabetic effects. This work was conducted to investigate the effects of the combination of GLP-1 and chamomile oil on differentiation of mesenchymal stem cells (MSCs) into functional insulin-producing cells (IPCs).

**Materials and Methods:**

In this experimental study, adipose MSCs derived from the adult male New Zealand white rabbits were assigned into four groups: control (without any treatment); GLP-1 (in which cells were treated with 10 nM GLP-1 every other day for 5 days); chamomile oil (in which cells were treated with 100 ug/ml *Matricaria chamomilla* L. flower oil every other day for 5 days); and GLP-1+ chamomile oil (in which cells were treated with 10 nM GLP-1 and 100 µg/ml *M. chamomilla* flower oil every other day for 5 days). Characterization of isolated MSCs was performed using flow cytometry, Alizarin red S staining and Oil red O staining. The expressions of genes specific for IPCs were measured using reverse transcriptase-polymerase chain reaction (RT-PCR) assay. Measurement of insulin and the cleaved connecting peptide (C-peptide) in response to different concentrations of glucose, were performed using ELISA kits.

**Results:**

Our results demonstrated that isolated cells highly expressed MSC markers and were able to differentiate into osteocytes and adipocytes. Additionally, using GLP-1 in combination with chamomile oil exhibited higher levels of IPCs gene markers including NK homeobox gene 2.2 (*NKX-2.2*), paired box gene 4 (*PAX4*), insulin (*INS*) and pancreatic duodenal homeobox-1 (*PDX1*) as well as insulin and C-peptide secretion in response to different glucose concentrations compared to GLP-1 or chamomile oil alone (P<0.05).

**Conclusion:**

Collectively, these findings establish a substantial foundation for using peptides in combination with natural products to obtain higher efficiency in regenerative medicine and peptide therapy.

## Introduction

Type 1 diabetes mellitus (Fig T1DM) is an autoimmune disease that is responsible for about 5-10% of all cases of diabetes around the world (1). During T1DM, initiation of chronic inflammatory responses gives rise to apoptotic and necrotic death of pancreatic ß-cells, and absolute insulin deficiency which, in turn, results in serious short-term and long-term side effects (2). It is urgent to discover new therapeutic options for treatment of T1DM and other degenerative diseases considering their high rate of morbidity and mortality (3-6). In recent years, stem cell-based therapy has been regarded as a promising strategy to treat immune-mediated diseases such as T1DM (7). Unique properties of mesenchymal stem cells (MSCs) including modulation of immune response, differentiation plasticity, easy attainability, and ability for inhibition of key factors involved in initiation of autoimmune disorders, make them excellent candidates to treat T1DM (8, 9). Although MSCs have demonstrated safety and efficacy in treatment of immune-mediated diseases such as T1DM, several drawbacks such as differentiation into undesired cells and migration to other body organs might limit their clinical applications (10). 

Glucagon-like peptide-1 (GLP-1) is an incretin hormone and food intake acts as a potent stimulator of its secretion by intestinal cells. GLP-1 plays an important role in a large number of physiological processes such as modulation of gastric emptying, blood glucose level, insulin secretion, glucose metabolism and appetite (11). Some previous studies have shown that GLP-1 might promote the growth and differentiation of ß-cells. For example, Abraham et al. (12) reported that GLP-1 contributed to the differentiation of nestinpositive islet-derived progenitor cells, present in the ducts and islet of the pancreas, into insulin-producing cells (IPCs). They concluded that GLP-1 exerted this function through alterations of gene expression profile. In fact, GLP-1 increased the expression of *PDX-1* and insulin promoter factor (*IPF-1*) gene. Moreover, previous reports have shown that natural products may exert therapeutic effects by targeting different cellular signaling pathways (13-15). Likewise, it is well-documented that natural products can enhance proliferation and differentiation of stem cells into desired cells (16). Chamomile (*Matricaria chamomilla* L.) is one of the well-documented medicinal herbs that belong to the Asteraceae (Compositae) family. Antioxidant and therapeutic properties of chamomile are due to the presence of terpenoids and flavonoids in its flowers (17). Some previous studies have shown that active ingredients of chamomile such as coumarins, quercetin, apigenin, and luteolin can reduce diabetes risk factors (18, 19). According to the aforementioned researches, we examined possible synergistic effects of GLP-1 and *M. chamomilla *L. oil on differentiation of MSCs into IPCs and their potential mechanisms. 

## Materials and Methods

### Reagents 

GLP-1, Collagenase type I, and *Matricaria chamomilla* L. flower oil were purchased from Sigma (Sigma-Aldrich Chemical, USA). Dulbecco’s modification of Eagle medium (DMEM/F12) and fetal bovine serum (FBS) were obtained from Gibco Company (USA). Rabbit Insulin ELISA Kit was purchased from Crystal Chem. Company (Crystal Chem. Inc., Downers Grove, IL). cDNA Synthesis Kit was supplied by EURx Company (Gdansk, Poland). SYBR® Premix Ex Taq ™ II (TliRNaseH Plus, RR820Q) was purchased from Takara company (Japan). Rabbit C-peptide ELISA Kit was purchased from Mybiosource Company. 

### Animals 

In this experimental study, male New Zealand white rabbits with a mean weight of 2.5 kg, were obtained from Razi Institute, Iran. All procedures and experimental tests were approved by the Animal Ethics Committee of Shahid Beheshti University of Medical Sciences (reference No. 1392. 49270). Rabbits were maintained in a temperature-controlled chamber set at 25 ± 1°C, with 12/12-hour light/dark cycles. They were fed with standard pellet chow and water ad libitum. After surgery and isolation of cells, the animals were permitted to recover spontaneous breathing and placed in their cage with free access to food and water. 

### Isolation of adipose-derived mesenchymal stem cells 

Rabbits were anesthetized intraperitoneally (IP) using ketamine (40 mg/kg) and xylazine (5 mg/kg). A midline incision was made in abdominal region. Approximately, 100 ml of adipose tissue was dissected from the perivisceral area. The adipose tissue was divided into small pieces in cold phosphate-buffered saline (PBS, Biochrom, Germany, pH=7.4). Then, small pieces of adipose tissue were homogenized and centrifuged at 175 g for 5 minutes. After removing supernatant, pellet was digested using 0.1% collagenase type I at 37°C under continuous shaking for 60 minutes. Then, the cell suspension was centrifuged at 175 g for 5 minutes. The supernatant was removed, and pellets were resuspended in an appropriate volume of the DMEM (Gibco, USA) supplemented with 10% FBS, and 1% penicillin-streptomycin and incubated at 37°C in a humid incubator with 5% CO_2_ to acquire enough cell density. 

### Identification of mesenchymal stem cells

To determine cell surface antigen profile of MSCs, fluorescence-activated cell sorting (FACS) was performed. In brief, after trypsinizing and washing with cold PBS containing 1% fetal calf serum (FCS), cells were incubated for 30 minutes with 10 µg/ ml antibodies in PBS per 1×10^6^ cells at 25°C in the dark. Antibodies applied in this work included CD45FITC, CD34-FITC, CD105-PE and CD73-PE (Dako, Denmark). To determine nonspecific fluorescence, cells were incubated with the isotype-matched antibody. A flow cytometer (Partec Pas III, Germany) was used to quantify the results. 

### Evaluation of osteogenic and adipogenic differentiation 

To evaluate adipogenic differentiation, Oil red O staining was performed. MSCs were incubated in a medium including 100 µg/ml 3-isobutyl1- methylxanthine, 10 µg/ml insulin, 10^-6^ M dexamethasone, 50 µM indomethacin in alpha- MEM medium supplemented with 10% FBS, for 3 weeks. To determine osteogenic differentiation, cells were incubated with a medium including 10 mM glycerophosphate disodium, 10^-7^ M dexamethasone, 50 µg/ml ascorbic acid in alpha-MEM medium supplemented with 10% FBS, for 4 weeks. Alizarin red S staining was used to observe calcium deposits. 

### Study design 

MSCs were cultured at a density of 1.5×10^6^ cells/ mL in alpha-MEM medium supplemented with 10% FBS containing 20 ng/ml of basic fibroblast growth factor (bFGF) and epidermal growth factor (EGF). Cells were randomly divided into the following four groups of 12 flasks in each. For control groups, cells did not receive any treatment (control). GLP-1 group only received 10 nM GLP-1 every other day for 5 days. Chamomile oil group only was treated with 100 µg/ml *Matricaria chamomilla* flower oil every other day for 5 days. GLP-1+chamomile oil group was treated with10 nM GLP-1+100 µg/ml *M. chamomilla* L. flower oil every other day for 5 days. 

### Reverse transcription polymerase chain reaction

Qiagen RNeasy kit (Qiagen Company, Valencia, CA, USA) was used to extract total RNA from 1×10^6^ differentiated cells following the manufacturer’s instructions. RNA concentration was determined using NanoDrop Microvolume Spectrophotometer and stored at -80°C. Then, total RNAwas converted into cDNA following the manufacturer’s protocol using a Dart cDNA kit. Quantitative polymerase chain reaction (PCR) was carried out using SYBR® Premix Ex Taq ™ II on a Rotor-Gene Q 5plex System (30-40 cycles). ß-actin was used as the internal control. The expression levels of each target gene was normalized against the internal control expression using 2^-ΔΔCt^ method. Reverse transcription- PCR (RT-PCR) primer pairs are shown in Table 1. 

### Assessment of insulin/C-peptide release 

To evaluate C-peptide release, we used Rabbit C-Peptide ELISA Kit. Measurement of insulin levels in culture media was performed using rabbit insulin ELISA kit. First, cells were pre-incubated with Krebs- Ringer buffer at 37°C for 2 hours. Then, cells were incubated with Krebs-Ringer buffer containing different doses of glucose (0, 15, and 30 mM) at 37°C for 1 hour. Finally, culture media was collected and assessments were performed. 

### Statistical analysis

All the data were presented as mean ± SD. GraphPad Prism software version 5.0 (CA, USA) was employed to analyze data. Values were subjected to a one-way analysis of variance (ANOVA) followed by Tukey multiple comparison tests. P<0.05 was accepted to be statistically significant. 

## Results

### Characterization of mesenchymal stem cells 

Three days after initial plating, we found that MSCs possess fibroblast-like morphology. Fourteen days after the initial plating, a confluent monolayer of MSCs was formed. Flowcytometric analysis demonstrated that CD105 (MSC marker) was expressed in 95.76% of cultured MSCs. Additionally, CD73 (MSC marker) was expressed in 96.86% of MSCs. 

**Fig.1:**
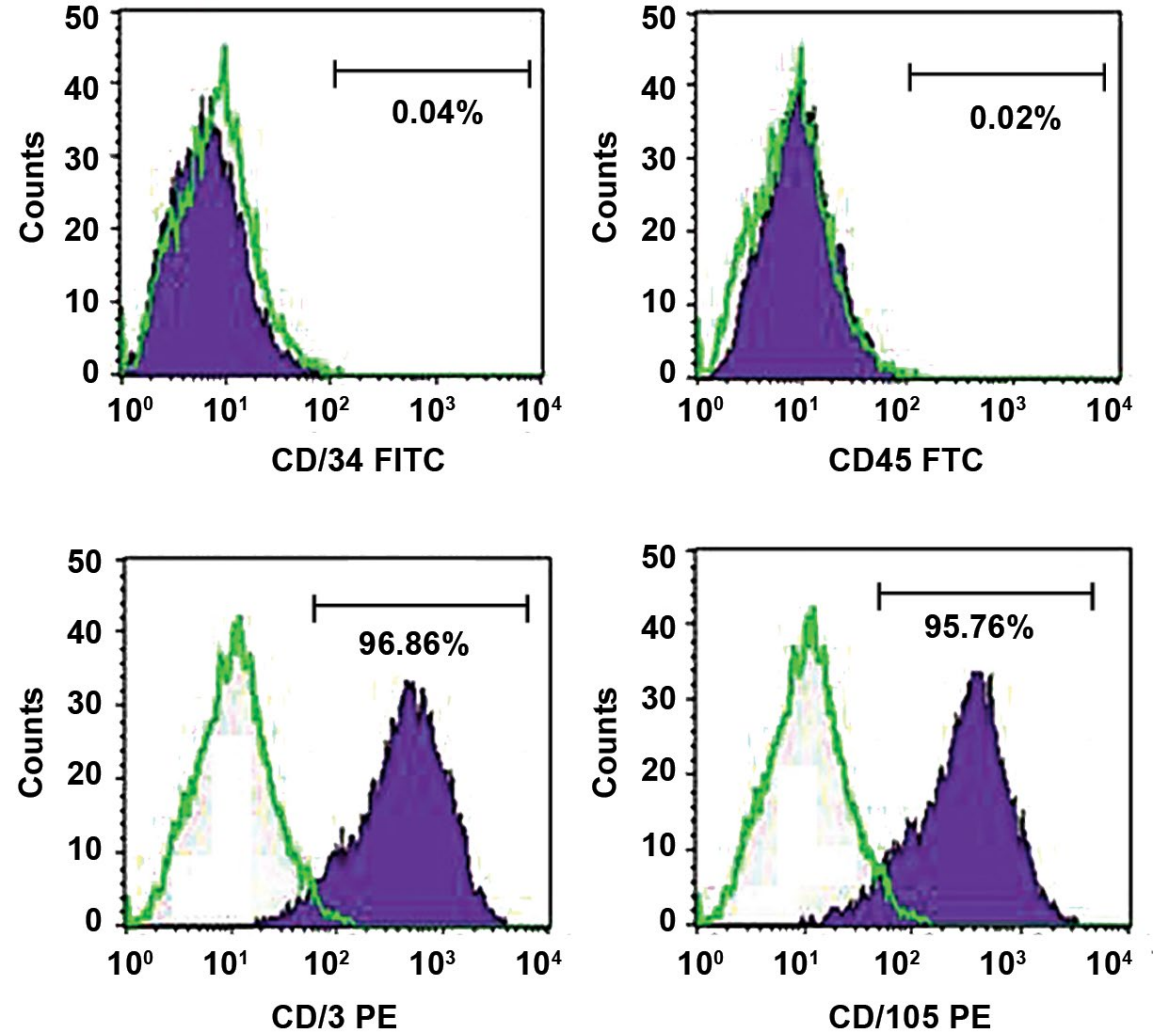
Immunophenotypic characterization of adipose-derived cells. The expressions of mesenchymal stem cell (MSC) markers such as CD73-PE and CD 105 PE, were higher than those of the hematopoietic progenitor marker CD34 and the pan-leukocyte marker CD45.

The hematopoietic progenitor marker CD34 (expressed in 0.04% of MSCs) and the pan-leukocyte marker CD45 (expressed in 0.02% of MSCs) did not indicate significant expression levels (Fig .1). 

### Osteogenic and adipogenic differentiation 

**Fig.2:**
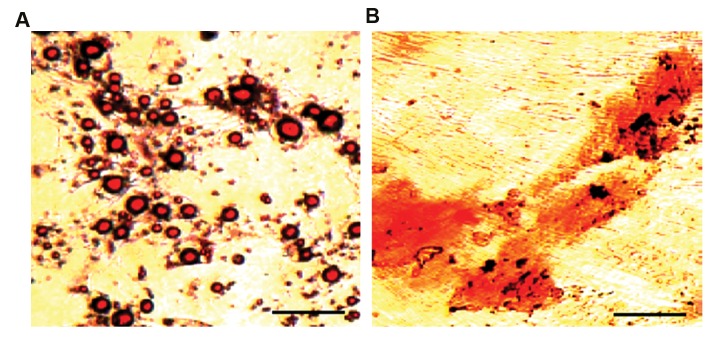
Evaluation of differentiation ability of mesenchymal stem cells (MSCs). **A.** Oil red O staining confirmed post-differentiation lipid accumulation in cultured cells and **B.** Alizarin red S staining showed mineralization and formation of calcium deposits in MSCs (scale bar: 100 µm).

Oil red O staining demonstrated that isolated MSCs have the ability to differentiate into adipocytes (Fig .2A). Alizarin red S staining showed the ability of the isolated MSCs for mineralization and formation of calcium deposits. These findings confirmed that isolated MSCs are able differentiate into osteocytes (Fig .2B). 

**Table 1:** Primer sequences for reverse transcription polymerase chain reaction (RT-PCR) analysis


Gene	Primer sequence (5ˊ-3ˊ)	Accession number	Sequence detected (bp)

NKX-2.2	F: GGGGTTTTCAGTCAAGGACA	XM_002710941.3	246
	R: AGTCCGTGCAGGGAGTATTG		
PAX4	F: GGGCTCTTTGTGAATGGCCG	XM_008258399.1	108
	R: TACCTTAAGGCTCCGGGAGAT		
INS	F: TCCTGCCCCTGCTGGC	NM_001082335.1	312
	R: AGTTGCAGTAGTTCTCCAG		
PDX1	F: AAAGCTCACGCGTGGAAAGG	XM_008275214.2	175
	R: TCAACATGACAGCCAGCTCCA		
β-actin	F: AAGATGACCCAGATCATGT	NM_001101683.1	188
	R: AGGTCCAGACGCAGGATG		


### The effects of GLP-1 and chamomile oil on morphology 1 and chamomile oil into IPCs, we measured mRNA of cultured MSCs 

**Fig.3:**
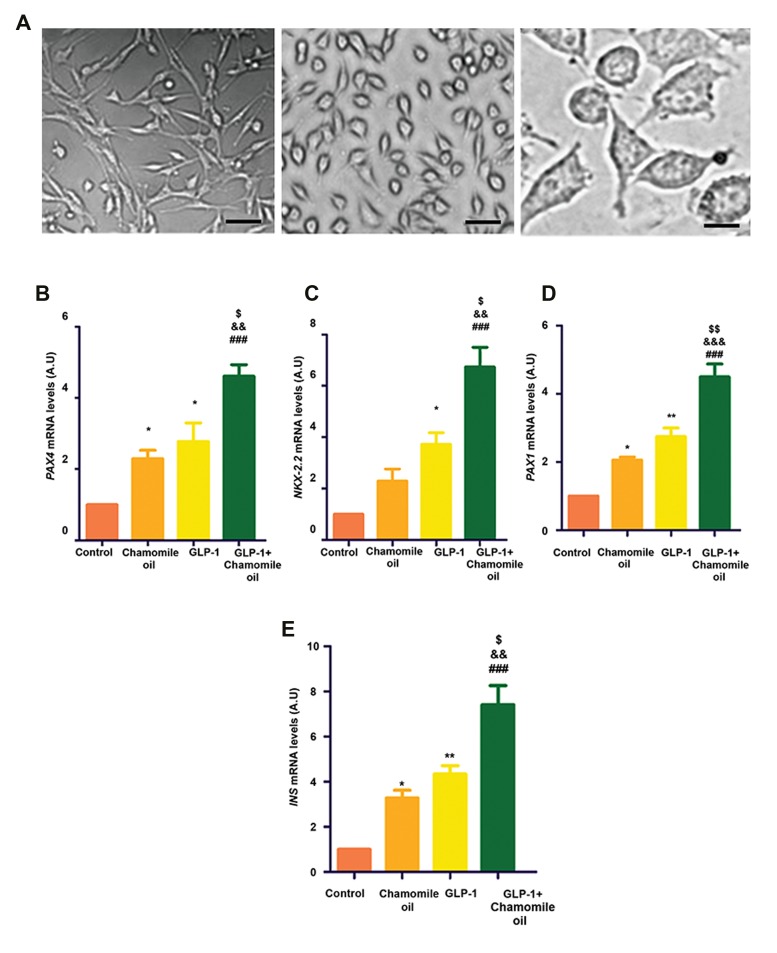
The effects of GLP-1+chamomile oil on cell morphology and gene markers of IPCs. **A.** The effects of GLP-1+chamomile oil on morphology of cells after 5 days. a. Control (scale bar: 100 µm), b and c. Presentation of cells treated with GLP-1+chamomile oil for 5 days at low and high magnifications (scale bars: 100 µm and 20 µm, respectively). The effects of GLP-1+chamomile oil on the expression of gene markers of insulin-secreting cells including: **B.** *PAX4*, **C.** *NKX-2.2*, **D.** *PDX1*, and **E.*** INS*. GLP-1; Glucagon-like peptide-1, IPCs; Insulin-producing cells, *; P<0.05, **; P<0.01 versus control, ###; P<0.001 versus the control, &&; P<0.01, &&&; P<0.001 versus chamomile oil, $; P<0.05, and $$; P<0.01 versus GLP-1.

The cells treated with GLP-1 and chamomile oil exhibited changes in their morphology. These cells were more flattened compared with control after 5 days, suggesting their differentiation into IPCs (Fig .3A).

### The effects of GLP-1 and chamomile oil on differentiation of MSCs into IPCs

To confirm differentiation of cells treated with GLP-1 and chamomile oil into IPCs, we measured mRNA levels of *NKX-2.2, PAX4, INS* and *PDX1* using RTPCR assay. Our results demonstrated that although cells treated with GLP-1 and cells treated with chamomile oil significant expressed *NKX-2.2, PAX4, INS* and *PDX1*, the expression of these IPCs markers was higher in cells treated with GLP-1+chamomile oil group (Fig .3B-E).

### The effects of GLP-1 and chamomile oil on the cleaved C-peptide levels in culture media 

**Fig.4:**
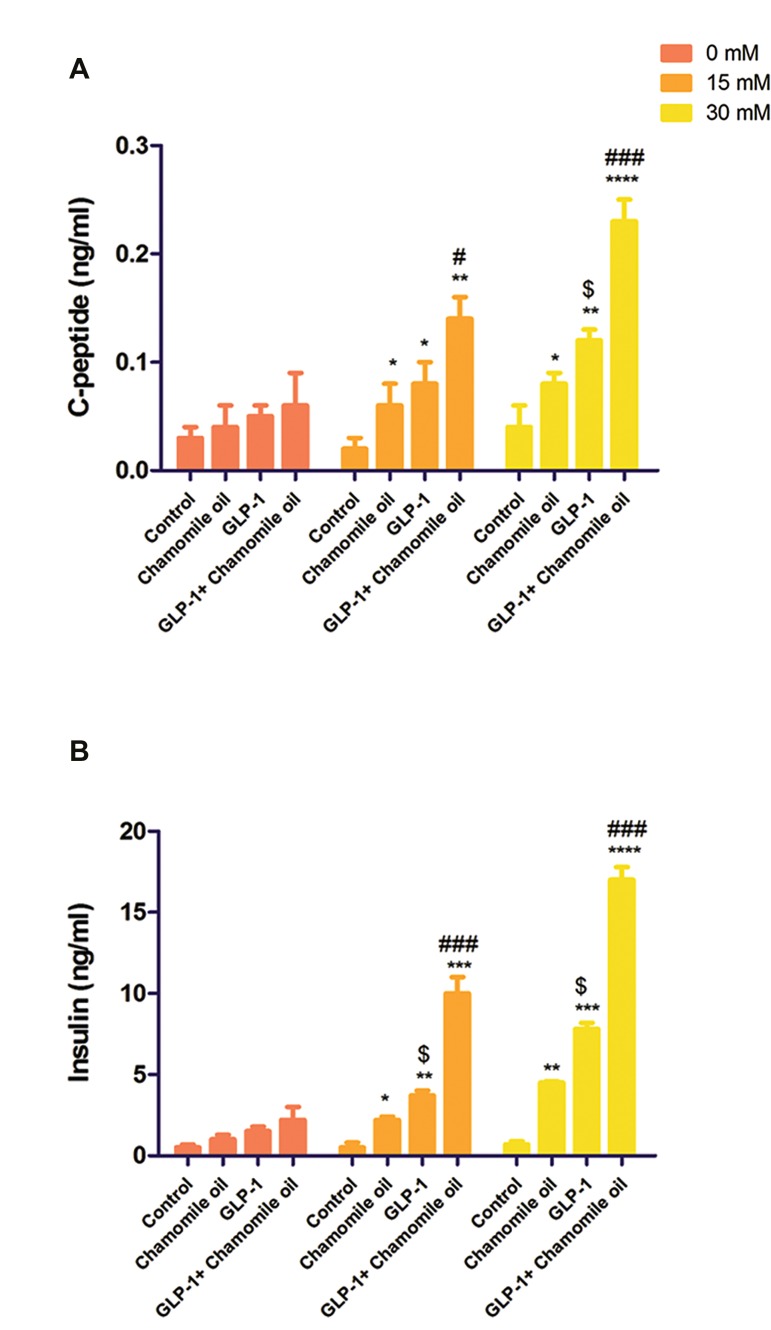
The effects of GLP-1 and chamomile oil on C-peptide and insulin levels inculture media. **A.** C-peptide level and **B.** Insulin level in culture media. GLP-1; Glucagon-like peptide-1, *; P<0.05, **; P<0.01, ***; P<0.001, ****; P<0.0001 versus control, $; P<0.05 versus chamomile oil, #; P<0.05, and ###; P<0.001 versus chamomile oil or GLP-1 alone.

To evaluate the function of treated cells, we measured C-peptide secretion by cells in response to different concentrations of glucose. As shown in Figure 4A, no significant differences were found among different groups in the absence of glucose (0 mM). Significant differences were observed in response to 15 and 30 mM concentrations of glucose. GLP-1+ chamomile oil group exhibited higher C-peptide secretion than cells treated either with chamomile oil alone or GLP-1 alone.

### The effects of GLP-1 and chamomile oil on insulin levels in culture media 

There were no significant differences among different groups in the absence of glucose (0 mM). Compared with other groups, GLP-1+chamomile oil showed the highest insulin secretion in response to 15 and 30 mM concentrations of glucose (Fig .4B).

## Discussion

In this work, we demonstrated that using peptide therapy and natural products together can produce synergistic effects on differentiation of MSCs into IPCs. In recent years, GLP-1, a peptide produced by dipeptidyl peptidase-4 (DPP4) cleavage of the gut incretin hormone, has attracted tremendous attention from scientific community for T1DM therapy because it can act as a growth factor to increase mass expansion of ß-cells and subsequently, insulin secretion. In fact, it is well known that this peptide promotes survival and proliferation of ß-cells (20). However, some recent studies have shown that GLP-1 facilitated the formation of new mature ß-cells (neogenesis) in the adult pancreases (21). Moreover, many previous reports have demonstrated that chamomile oil possesses many active ingredients that act as anti-diabetic, antioxidant, anti-inflammatory and antibacterial agents (22-24). For example, luteolin, a bioactive compound present in chamomile oil, increases insulin secretion and activates adipokines/cytokines in adipocytes through induction of the peroxisome proliferator-activated receptor-γ (PPARγ) pathway (25, 26). 

In this study, we investigated the synergistic effect of GLP-1 and chamomile oil on differentiation of MSCs into insulin-secreting cells. The isolated MSCs exhibited an increased expression of MSCs markers, whereas they did not demonstrate a significant expression of the hematopoietic progenitor and pan-leukocyte markers. These findings confirmed a highly purified MSC population. In agreement with the results of the present study, Razavi Tousi et al. (27) reported that the isolated MSCs strongly expressed MSCs marker CD105, but not CD 45 and CD34. On the other hand, isolated cells were able to differentiate into osteocytes and adipocytes. In agreement with this study, a previous report showed that the isolated MSCs can be differentiated into osteocytes and adipocytes (28). Furthuremore, a previous study indicated that addition of GLP-1 to the culture media of mouse embryonic stem cells, contributed to differentiation into IPCs (29). 

To examine the synergistic effects of GLP-1 and chamomile oil, we measured mRNA levels of *PAX4* and *NKX-2.2*. The activity of homeodomain protein *NKX-2.2* and the NK-family members is necessary for differentiation and the maturation of ß-cells. It seems that *NKX-2.2* contributes to differentiation of ß-cells through interaction with *PAX4*. Loss of *PAX4* results in dowregulation of *INS, PDX1* and HB9 in ß-cell precursors (30). Our findings showed that using peptide and chamomile oil significantly increased mRNA levels of *PAX4* and *NKX-2.2* compared to control, GLP-1 group only and chamomile oil only treated groups. Recent studies have shown that expression of *PDX1* is necessary for maintaining ß-cell identity and function via suppression of a-cell program (31). To examine whether GLP-1 and chamomile oil can contribute to formation of ß-cells and maintain their function, we also measured mRNA levels of *PDX1* and *INS*. Our findings showed that although both peptide and chamomile oil administered alone, increased the mRNA levels of *PDX1* and *INS* in cultured cells, the effects of their co-administration was higher than single treatments. Consistent with the present study, increased mRNA levels of *NKX-2.2, PAX4, PDX1, INS* were found after differentiation of human embryonic stem cells (hESCs) into IPCs during a sevenstage protocol (32). The cleaved C-peptide is a byproduct and a hallmark of average daily insulin production. To form mature insulin hormone, a single-chain proinsulin peptide is translated and then converted into C-peptide and disulfide-linked insulin (33). It has been reported that C-peptide secretion of IPCs derived from hESCs in response to 15 mM glucose was about 0.15 ng/ml after 33 days (34). Compared with this report, the present study showed that C-peptide secretion of MSCs treated with chamomile oil+GLP-1 in response to 15 mM glucose was about 0.15 ng/ml after 5 days. Likewise, differentiated cells exhibited higher insulin secretion in response to higher concentrations of glucose. Other studies also indicated that IPCs derived from embryonic stem cells displayed higher insulin secretion in response to higher concentrations of glucose (35). Additionally, the highest insulin levels in culture media were found in chamomile oil+GLP-1 group. The cells treated with peptide and chamomile oil exhibited more flattened morphology. Consistent with our study, Abraham et al. (12) reported that differentiation of human pancreatic islet-derived progenitor cells into IPCs in the presence of GLP-1 resulted in more flattened morphology. Also, this research group reported that insulin concentration in media was about 2.4 ng/ml after treatment of nestin-positive isletderived progenitor cells (NIPs) with 10 nm GLP-1 for 7 days. Consistently, the present study demonstrated that insulin concentration in media of cells treated with 10 nM GLP-1 alone in the absence of glucose, was about 2.5 ng/ ml, whereas it was increased to 4-7 ng/ml in response to 15 and 30 mM glucose.

## Conclusion

Collectively, our finding demonstrated that chamomile oil in combination with GLP-1 more efficiently enhances the differentiation of adipose-derived MSCs into IPCs. These findings establish a substantial foundation for using peptides in combination with natural products to obtain higher efficiencies in regenerative medicine. 
